# The challenge of constructing an international XAFS database

**DOI:** 10.1107/S1600577518006963

**Published:** 2018-05-30

**Authors:** Kiyotaka Asakura, Hitoshi Abe, Masao Kimura

**Affiliations:** a Institute for Catalysis, Hokkaido University, 21-10 Kita, Sapporo, Hokkaido 001-0021, Japan; b Photon Factory, Institute of Materials’ Structure Science, High Energy Accelerator Research Organization, 1-1 Oho, Tsukuba, Ibaraki 305-0801, Japan; c Department of Materials’ Structure Science, School of High Energy Accelerator Science, SOKENDAI (The Graduate University for Advanced Studies), 1-1 Oho, Tsukuba, Ibaraki 305-0801, Japan

**Keywords:** XAFS database, automatic data deposition, data repository system

## Abstract

The present state of XAFS databases, particularly in Japan, and proposals for future directions are presented. International collaboration is important for enlarging the database for further development of XAFS spectroscopy.

## Abstract 2

The present situation of the XAFS database in Japan and a proposal for the construction of an international collaboration XAFS database are presented.

## 1. Why we need an international XAFS database   

X-ray absorption fine-structure (XAFS) spectroscopy is a powerful technique for determining local structure in metals with no long-range order (Iwasawa *et al.*, 2016 ▸). Because a continuum X-ray source is required, XAFS spectroscopy became a practical characterization technique only after synchrotron radiation sources were developed.

**Figure 1:**
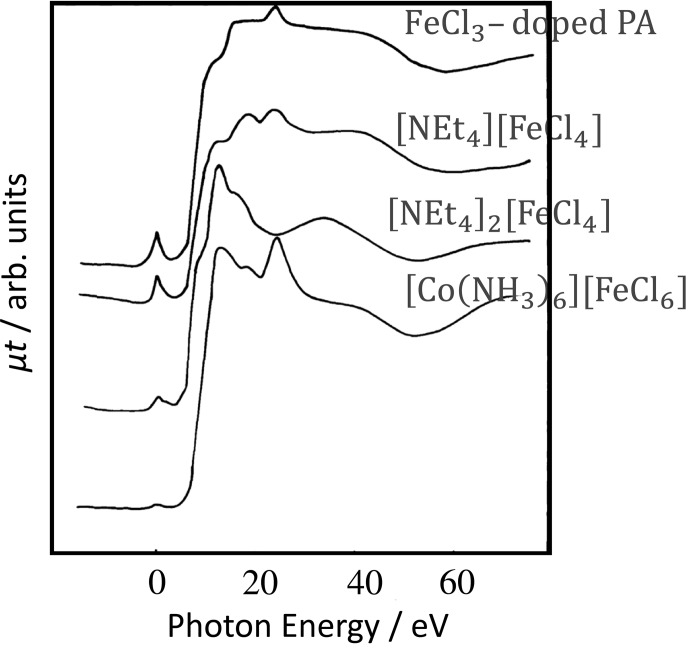
Fe *K*-edge XANES spectra of selected Fe chlorides. The pre-edge peak is set at 0 eV. The Fe chloride dopant was characterized by XAFS spectroscopy. By comparing with reference compounds, the Fe species in the PA could be identified as [FeCl_4_]^−^. μ and *t* are the absorption coefficient and thickness of the sample. [Reproduced from Asakura *et al.* (1985 ▸) with permission from the Chemical Society of Japan, Copyright (1985).]

Since the first measurements were made by Eisenberger at the Stanford Synchrotron Radiation Lightsource (Kincaid & Eisenberger, 1975 ▸), large quantities of XAFS data have been collected. These data can be divided into two groups: (1) reference compounds with known structures and properties, and (2) unknown target materials under investigation. The data for the former group are important for materials scientists who wish to analyse the XAFS spectra of unknown compounds by comparing them with those of reference compounds. Fig. 1 ▸ shows the XAFS spectra of different Fe chloride compounds. Some compounds produce a remarkable pre-edge peak that can be assigned to a 1*s*-to-3*d* transition, which is dipole forbidden, though it becomes stronger when centrosymmetry is broken in a tetrahedral structure (Asakura *et al.*, 1985 ▸; Shulman *et al.*, 1976 ▸). Thus, the Fe chloride species in FeCl_3_-doped polyacetyl­ene (PA) can be identified by comparison with XAFS spectra of reference compounds. A collection of XAFS spectra may be especially important when XAFS spectroscopy is performed by non-specialists. Collections of typical XAFS spectra are published in paper form, *e.g.* books. The Catalysis Society of Japan has published the *Catalyst Handbook*, which includes XAFS spectra of reference compounds containing catalytically important elements (Catalysis Society of Japan, 2008 ▸).

**Figure 2:**
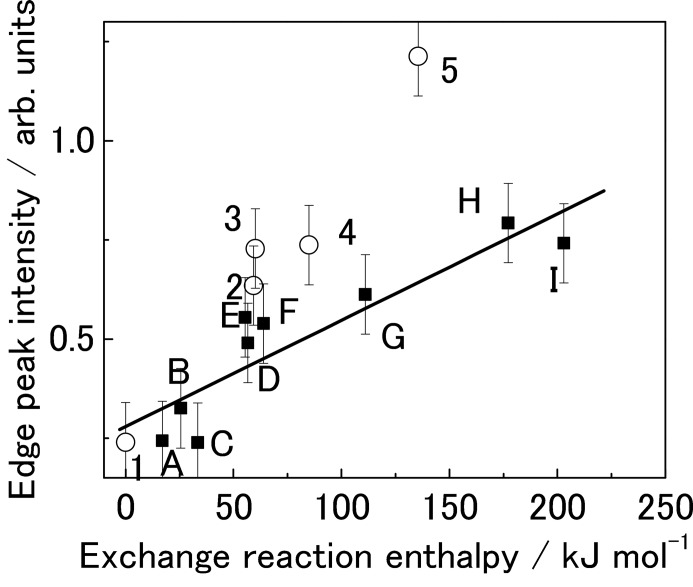
Relationship between edge peak intensity for Ag (I) compounds and covalence (Miyamoto *et al.*, 2010 ▸, 2012 ▸). [Reproduced from Miyamoto *et al.* (2010 ▸) with permission, Copyright (2010), American Chemical Society.]

An open-access Internet-based database of XAFS spectra in digital form is more convenient for all researchers to update and search. Such a large comprehensive database would be useful not only for XAFS non-specialists but also for specialists who want to elucidate general concepts from XAFS spectra. Fig. 2 ▸ shows the relationship between the intensity of the edge-peak of Ag^+^ *L*_3_ X-ray absorption near-edge structure (XANES) and the covalence of Ag compounds (Miyamoto *et al.*, 2010 ▸, 2012 ▸), which demonstrates a positive correlation. Such examples can be found in other studies, such as Lytle’s white-line analysis and Wong’s V oxide pre-edge and edge-shift analysis (Lytle *et al.*, 1979 ▸; Wong *et al.*, 1984 ▸). Eventually, deep learning methods could derive such relations easily and automatically if a large database of XAFS spectra was available and related to other material databases (Takigawa *et al.*, 2016 ▸).

## 2. Overview of currently available XAFS databases   

Several databases of XAFS spectra have been developed, as shown in Table 1 ▸. These databases are free, and they generally permit open access. The F. W. Lytle database has a long history, and is the largest and perhaps most well known XAFS database. The SPring-8 BL14 database, which requires a user ID and password to view and search data, has ample data (more than 800) for reference compounds.

Recently, ‘Photon Beam Platform’, a national project in Japan to promote the collaboration among synchrotron radiation and laser facilities, has been carrying out round-robin tests of XAFS data in the data format of XML (eXtensible Markup Language). An international proposal of round-robin tests of XAFS data has also been proposed (Chantler *et al.*, 2018 ▸).

This paper focuses on the XAFS database developed as a collaboration between the Hokkaido University Institute for Catalysis (ICAT) and the Japanese XAFS Society (JXS). We would like to propose an international collaboration to make a new big and open database for XAFS.

## 3. ICAT XAFS database in Japan   

### 3.1. History   

The Hokkaido University ICAT started to build the XAFS database in 2011 as a project of the Joint Usage/Research Center for catalysis, in collaboration with JXS. The database is accessible to anyone through the Internet. Volunteers working in the XAFS field can register for an ID and password to upload their data to the database. For security and traceability, data files contain the name of the person who uploaded the data. The website is managed by ICAT.

### 3.2. Fundamental ICAT data policies   

The basic policies of the ICAT XAFS database are as follows:

(i) Volunteer base. Anyone who is registered can upload their data.

(ii) Open-access database. Anyone can see and use the data. They are asked to include a citation in published work if they used the data.

(iii) Simple dataset format. The data have a text-based format, and only the energy and μ*t* of the raw spectrum are recorded, in addition to some metadata.

(iv) Compatibility. Foil XAFS data that are measured at the same beam time and the same beamline are deposited simultaneously to make energy calibration possible. Consequently, we can compare the data measured at different beam times, beamlines and facilities.

(v) Target. Dataset includes primarily standard compounds with known structures.

(vi) Guarantee of accuracy. A ‘majority rule’ approach is used to improve accuracy. The database allows deposition of multiple records for the same compounds.

**Figure 3:**
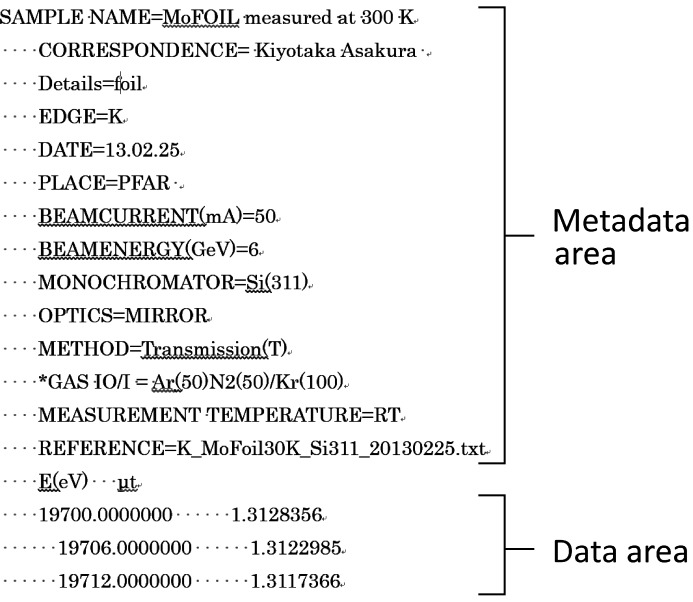
ICAT data file structure.

Each data file has a metadata area and a data area, as shown in Fig. 3 ▸. Table 2 ▸ lists the available metadata fields in the ICAT database. Although detailed metadata information is helpful, too many required inputs will reduce the motivation of volunteers, so the number of metadata fields are kept as small as possible.

One of the most important policies is compatibility. Although the absolute angle of each monochromator can be determined by Pettifer’s method or other (Pettifer & Hermes, 1985 ▸), it is more convenient to calibrate each spectrum using foil measured at the same time. The data are uploaded with the foil data measured at the same time. Uploading foil data helps us to obtain the signal-to-noise ratio (S/N) and the energy resolution of the XAFS measurement systems by comparison with the foil XAFS data in addition to adjusting the energy scale.

Another important policy is the guarantee of accuracy, that is, the quality of each XAFS spectrum. One way to guarantee accuracy is to permit only highly qualified researchers to measure spectra under a strict, specific protocol and upload the data in a rigorously controlled way. However, this would require more qualified researchers, time and money that are not generally available. Also, the amount of data stored per day would be limited and it would be difficult to construct a large database.

ICAT uses another strategy for data quality, that is, majority rule. The volunteers, who are expected to have average skill for measuring XAFS spectra and preparing datasets, measure the spectra and upload the data with the metadata. When many volunteers randomly and independently upload spectra, eventually different versions of the same reference spectra are collected. These data points are statistically distributed so that the average spectra and their error bars can be determined. Data that lie within the error bars are reliable, but a spectrum lying outside this range is either wrong or represents a different phase. Thus, multiple spectra of the same compound improve the quality of the database. Once several spectra of the same compound are available, users check the accuracy by comparing the spectra of the same compound by themselves.

## 4. Problems with the existing ICAT database   

Despite its usefulness, the ICAT database has several drawbacks. The first problem is the small number of data files in the database. This makes it difficult to apply the majority rule and derive effective relationships between spectral features and material properties. A way is needed to obtain more data effectively. The second problem is that the database is not linked to other material databases, such as X-ray diffraction, optical spectra, and other physical and chemical properties. The third problem is the format. The ICAT database uses a simple text-base format. Several data formats have been proposed, such as CIF XAFS, XDI (eXtensible Data Interchange), JASON (JavaScript Object Notation), NeXus and HDF5 (Ascone *et al.*, 2012 ▸; Ravel & Newville, 2016 ▸; Könnecke *et al.*, 2015 ▸). The NeXus and HDF5 formats can store and manage imaging data written in a binary format. Adoption of NeXus or HDF5 may be a good choice for a flexible database, but it must first be designed well after long deliberation. Nevertheless, the text format with only energy and μ*t* data is simple, easy to implement, and transferrable to other formats. Although continued discussion of the XAFS database format is needed, it might be expedient to start database construction in a text format and convert the data to a unified format later.

## 5. Future database development: bigger is better   

In the future, computers will be able to automatically evaluate the correctness of spectra based on the majority rule. Even if information about a sample is inadequate, computers could classify the samples into several subsets. For example, if the sample name is entered only as TiO_2_, the spectra would need to be associated with one of the three different crystalline forms of TiO_2_: rutile, anatase and brookite. Thus, the computer would need to assign the spectra to one of these crystal forms using other spectra data. Some spectra may be located between two of the groups, indicating a mixture, so it is better to have more data of the same compounds available for comparison. A large database will reduce the probability of misinterpretation and improve our understanding of the spectra. The future direction of the XAFS database is to be bigger and more open. The majority rule, in combination with advanced computing, such as artificial intelligence (AI), can work to reinforce data quality and reliability and to elucidate meaningful new concepts through deep learning. The problem is how to construct a larger database as mentioned above. Since we collect data manually at the moment, the ICAT database only contains a limited number of data files. If the data were automatically stored in an appropriate way, all measured data could be included in the database. Manual input would be limited to metadata and clarification and categorization of data into spectra for reference or unknown compounds.

Since most XAFS spectra are measured at synchrotron radiation facilities, we propose the following system:

(i) Store all data measured at these facilities automatically in the facility’s computer in appropriate formats and with metadata. Thus, each facility can construct the larger database.

(ii) Then combine the databases from each facility by connection to the internet. Before that, however, we need to decide on rules for data disclosure. The fundamental principle should be that all scientific data must be open to the public eventually, but the data owner rights and originality should be reserved.

Using these basic ideas, the data can be made accessible to the public but only after the agreement of the data owners. This will allow data owners to choose to keep their data confidential, but it is hoped that they would release the data after the associated studies are published in the literature.

Data disclosure would be beneficial to our society in two ways: (1) the quantity of data in the XAFS database would increase, improving its quality and accessibility for everyone, and (2) the quality and transparency of published research would be improved by disclosing the raw data. The cooperation of publishers would be important, and it is hoped that they would require disclosure of the raw XAFS data when a paper is accepted. Facilities could provide further support by making data disclosure contracts with all users before they start experiments. The raw data should also be made available when a patent is established. In addition to these rules, all data should be disclosed after a certain period (ten years for example). Facilities should retain responsibility for data security and protection; otherwise, users would not allow facilities to store their data.

One more important suggestion is to define a unique digital data number, similar to the digital object identifier (DOI) for a published paper. A data identifier would allow the data to be cited when it is used in a publication. This would increase the incentive for owners to upload and to open their data. Like the citation index for a paper, a user could benefit from the number of citations of their data. This would provide further motivation to perform precise measurements and develop higher-quality data. A suggested format for the data identifier is as follows:
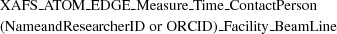
Note that the identifier must be automatically determined and unique.

The linkage of facility databases will be another important task, since XAFS data are not standalone. To derive structure–property relationships, XAFS data need to be considered in the context of the physical and chemical properties of the measured material. To do this, a clear sample name must be determined uniquely and universally. If the sample is a standard reference compound, this should be clearly conveyed by some unique sample identifier. The Chemical Abstracts Service (CAS) number is the best choice for the sample identifier because it will be comprehensive and make it easier to associate the sample with physical and chemical properties. Moreover, the majority rule will work more effectively because different files for the same material can be linked easily, possibly automatically. The facilities could encourage input of the CAS number into the metadata by providing a CAS number dictionary to users. The data owner should provide their own data name according to some rule, such as:
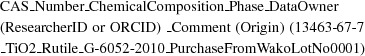



## 6. What to do next   

We believe that an open XAFS database is necessary but also very useful. Some issues should be set aside for now, and each facility should start to construct a database or repository of its own data, with the goal of eventual linkage to a larger database. We propose the following actions:

(i) Ask all facilities to start collecting XAFS data.

(ii) Decide on simple naming rules for linkage to other databases.

(iii) Decide on naming rules for data files, along the lines of the DOI.

(iv) Start the discussion for determining data release rules.

We hope this proposal can be discussed at the next Q2XAFS and at other meetings.

## Figures and Tables

**Table 1:** Available XAFS databases (as of 31 December 2017)

Database	URL	Features
F. W. Lytle database	http://ixs.iit.edu/database/	Text format with periodic table interface
		All data obtained by F. W. Lytle
		19307 records covering 74 elements

CARS	http://cars.uchicago.edu/xaslib/search	Text (XDI) format with periodic table interface and graphical output of spectra (Newville *et al.*, 2015 ▸)
		171 records covering 13 elements
		Measured at the Advanced Photon Source, SSRL and National Synchrotron Light Source
		Includes suites (tagging-related spectra) and user ratings

SPring-8 BL14	https://sp8dr.spring8.or.jp/portal/dspace	Only available to SPring-8 users
		725 records
		Compressed text file (ZIP) format with metadata that contains vendor and lot number

European Synchrotron Radiation Facility, ID21 group	http://www.esrf.eu/home/UsersAndScience/Experiments/XNP/ID21/php.html	Sulfur database
		41 (inorganic) and 26 (organic) records for S compounds
		Graphical output, but text format is available
		User name is included

Hokkaido University ICAT and JXS	https://www.cat.hokudai.ac.jp/catdb/index.php?action=xafs_login_form&opnid=2	209 records and 24 elements
		Text format with metadata
		Includes user name, beamline and facility
		Open access, but data uploading is restricted to users with ID and password

**Table 2:** Available metadata fields in the ICAT database

Sample name	Beam current (mA)
Structural details (powder, foil, solution)	Beam energy of storage ring (GeV)
Absorption atom	Higher harmonics rejection method
Edge	Focus
Reference sample (usually foil)	Detection method
Date of measurement	I_0_ and I gases if ionization chamber used
Monochromator (crystal)	Measurement temperature
Facility	Comments
Beamline	
